# Ischemia-induced cell depolarization: does the hyperpolarization-activated cation channel HCN2 affect the outcome after stroke in mice?

**DOI:** 10.1186/2040-7378-5-16

**Published:** 2013-12-27

**Authors:** Petra Ehling, Eva Göb, Stefan Bittner, Thomas Budde, Andreas Ludwig, Christoph Kleinschnitz, Sven G Meuth

**Affiliations:** 1 Department of Neurology, and Institute of Physiology, Neuropathophysiology, Albert-Schweitzer-Campus 1, Westfälische Wilhelms University, 48149 Münster, Germany; 2 Department of Neurology, University Clinic Würzburg, Josef-Schneider-Str. 11, Würzburg, Germany; 3 Department of Neurology, Albert-Schweitzer-Campus 1, Westfälische Wilhelms University, Münster, Germany; 4 Institute of Physiology I, Westfälische Wilhelms University, Robert-Koch-Str. 27a, Münster, Germany; 5 Institute of Experimental and Clinical Pharmacology and Toxicology, Friedrich-Alexander Universität Erlangen-Nürnberg, Fahrstr. 17, Erlangen, Germany; 6 Department of Neurology, ICB, Mendelstr. 7, 48149 Münster, Germany

## Abstract

**Background:**

Brain ischemia is known to include neuronal cell death and persisting neurological deficits. A lack of oxygen and glucose are considered to be key mediators of ischemic neurodegeneration while the exact mechanisms are yet unclear. In former studies the expression of two different two-pore domain potassium (K_2P_) channels (TASK1, TREK1) were shown to ameliorate neuronal damage due to cerebral ischemia. In neurons, TASK channels carrying hyperpolarizing K^+^ leak currents, and the pacemaker channel HCN2, carrying depolarizing I_h_, stabilize the membrane potential by a mutual functional interaction. It is assumed that this ionic interplay between TASK and HCN2 channels enhances the resistance of neurons to insults accompanied by extracellular pH shifts.

**Methods:**

In C57Bl/6 (wildtype, WT), *hcn2*^+/+^ and *hcn2*^-/-^ mice we used an *in vivo* model of cerebral ischemia (transient middle cerebral artery occlusion (tMCAO)) to depict a functional impact of HCN2 in stroke formation. Subsequent analyses comprise behavioural tests and *hcn2* gene expression assays.

**Results:**

After 60 min of tMCAO induction in WT mice, we collected tissue samples at 6, 12, and 24 h after reperfusion. In the infarcted neocortex, hcn2 expression analyses revealed a nominal peak of hcn2 expression 6 h after reperfusion with a tendency towards lower expression levels with longer reperfusion times. Hcn2 gene expression levels in infarcted basal ganglia did not change after 6 h and 12 h. Only at 24 h after reperfusion, hcn2 expression significantly decreases by ~55%. However, 30 min of tMCAO in hcn2-/- as well as hcn2+/+ littermates induced similar infarct volumes. Behavioural tests for global neurological function (Bederson score) and motor function/coordination (grip test) were performed at day 1 after surgery. Again, we found no differences between the groups.

**Conclusions:**

Here, we hypothesized that the absence of HCN2, an important functional counter player of TASK channels, affects neuronal survival during stroke-induced tissue damage. However, together with a former study on TASK3 these results implicate that both TASK3 and HCN2 which were supposed to be neuroprotective due to their pH-dependency, do not influence ischemic neurodegeneration during stroke in the tMCAO model.

## Background

Ischemic stroke occurs due to an interruption of blood supply to corresponding areas of the brain, initiating an ischemic cascade. The depletion of oxygen or glucose in ischemic brain tissue sets off a series of interrelated events that result in neurodegeneration. Consequently, this leads to a high rate of permanent disabilities and even death [1]. Generally, neurotoxicity can be mediated by ionic imbalances that contribute to apoptosis (programmed cell death). Many efforts have been spent so far on investigating neuronal ion channel function and regulation after stroke in different animal models [2-5]. Cells that undergo apoptosis have a strongly depolarized membrane potential prior to cell death [6,7]. In contrast, a hyperpolarized membrane potential has been reported to be an important mechanism promoting resistance to apoptosis [8,9]. Thus, an important indicator for neuronal survival seems to be the stability of the resting membrane potential. Among others HCN channels (hyperpolarization-activated and cyclic nucleotide-gated channels, also known as pacemaker channels) help to maintain a stable cell membrane potential at rest and thereby define the excitability of CNS neurons [10-13]. For thalamocortical relay neurons, it could be demonstrated that two ion channels, which are predominantly active at rest, strongly influence the resting membrane potential. The hyperpolarizing K^+^ leak current carried by two-pore domain K^+^ (K_2P_) channels is counterbalanced by a depolarizing I_h_ carried by HCN channels resulting in a stable resting membrane potential in thalamic neurons [14,15].

Interestingly, acidification, one initial pathophysiological event after arterial occlusion, inhibits both TASK [16-19] as well as HCN channels [20,21]. Thereby, the acidified milieu after arterial occlusion most probably influences the activity of acid-sensing ion channels as well as the cell membrane potential. Thus, a future therapeutic strategy to further stabilise the resting membrane potential of neurons might promote their survival in an early phase of stroke development.

The HCN channel family comprises four members (HCN1-4). Currents through HCN channels (I_h_) have unusual characteristics including activation upon hyperpolarization, permeability to K^+^ and Na^+^, as well as modulation by cyclic AMP [12]. Originally, they were identified as pace making channels in the heart that set cardiac rhythm [22-26]. Besides pacing the heart these channels are recognized as ubiquitous components of the nervous system. By setting the membrane potential and input resistance at rest, HCN channels play an important role to the integrative function and the sensitivity to synaptic inputs in neurons [12,24]. Channel malfunction could be linked to central diseases including epilepsy [13,27]. *Hcn2* transcripts were found at high levels nearly ubiquitously in brains of adult mice, and the strongest signals were seen in the olfactory bulb, hippocampus, thalamus and brainstem [28]. Here, we test the hypothesis that functional HCN2 channels limit the infarct volumes and improve neurological and motor abilities in a mouse model of stroke (tMCAO). Based on their inhibition by acidification which occurs during arterial occlusion one might predict that less active HCN2 channels favour a more hyperpolarized membrane potential and a reduced susceptibility to brain damage.

## Methods

### Real-time PCR

For *hcn2* gene expression ipsilesional neocortices and basal ganglia of C57Bl6 WT mice (Charles River, Sulzfeld, Germany) were analysed 6, 12 and 24 h after reperfusion together with corresponding tissue samples from sham-operated mice. Tissue homogenization, RNA isolation and real-time RT-PCR were performed as described [29]. Total RNA was prepared with a Miccra D-8 power homogenizer (ART) using the TRIzol reagent® (Invitrogen) and was quantified spectrophotometrically. Then, 1 μg of total RNA were reversely transcribed with the TaqMan® Reverse Transcription Reagents (Applied Biosystems) according to the manufacturer’s protocol using random hexamers. Relative gene expression levels of *hcn2* (assay ID: Mn00468538_m1, Applied Biosystems) was quantified with the fluorescent TaqMan® technology. GAPDH (TaqMan® Predeveloped Assay Reagents for gene expression, part number: 4352339E, Applied Biosystems) was used as an endogenous control to normalize the amount of sample RNA. The PCR was performed with equal amounts of cDNA in the StepOnePlusTM Real-Time PCR System (Applied Biosystems) using the TaqMan® Universal 2x PCR Master Mix (Applied Biosystems). Reactions (total volume 12.5 μl) were incubated at 50°C for 2 min, at 95°C for 10 min followed by 40 cycles of 15 sec at 95°C and 1 min at 60°C. Water controls were included to ensure specificity. Each sample was measured in triplicate and data points were examined for integrity by analysis of the amplification plot. The comparative Ct method was used for relative quantification of gene expression as described [30].

### Induction of cerebral ischemia

Animal experiments were approved by governmental agencies (Regierung von Unterfranken, Würzburg, Germany, approval number 54/09) for animal research and conducted according to the recommendations for research in mechanism-driven basic stroke studies [31], current ARRIVE guidelines (http://www.nc3rs.org/ARRIVE). Focal cerebral ischemia was induced in 8-20 weeks old *hcn2*^+/+^ and *hcn2*^-/-^[13] littermates of either sex weighing 15-30 g (*hcn2*^+/+^: 20-30 g; *hcn2*^-/-^: 15-25 g) by transient middle cerebral artery occlusion (tMCAO) as described previously [32,33]. Briefly, mice were anesthetized with 2.5% enflurane (Abbott, Wiesbaden, Germany) in a 70% N_2_O/30% O_2_ mixture. Core body temperature was maintained at 37°C throughout surgery using a feedback-controlled heating device. Following a midline skin incision in the neck, the proximal common carotid artery and the external carotid artery were ligated and a standardized silicon rubber-coated 6.0 nylon monofilament (6021; Doccol Corp., CA, USA) was inserted and advanced via the right internal carotid artery to occlude the origin of the right MCA. The intraluminal suture was left *in situ* for 0.5 or 1 hour, respectively. Then animals were re-anesthetized and the occluding monofilament was withdrawn to allow reperfusion. After 24 hours neurological deficits were scored by two blinded investigators and quantified according to Bederson [34]: 0, no deficit; 1, forelimb flexion; 2, as for 1, plus decreased resistance to lateral push; 3, unidirectional circling; 4, longitudinal spinning; 5, no movement. For the gript test, the mouse was placed midway on a string between two supports and rated as follows [35]: 0, falls off; 1, hangs onto string by one or both forepaws; 2, as for 1, and attempts to climb onto string; 3, hangs onto string by one or both forepaws plus one or both hind paws; 4, hangs onto string by fore- and hind paws plus tail wrapped around string; 5, escape (to the supports).

### Laser-Doppler flowmetry

Laser-Doppler flowmetry (Moor Instruments, Axminster, United Kingdom) was used to monitor cerebral blood flow in the right MCA territory (6 mm lateral and 2 mm posterior from bregma) [36] in *hcn2*^+/+^ and *hcn2*^-/-^ (n = 4/group) before surgery (baseline), immediately after MCA occlusion (ischemia), and 5 minutes after removal of the occluding monofilament (reperfusion).

### Determination of infarct size

Mice were sacrificed 24 hours after tMCAO. Brains were quickly removed and cut in 2 mm thick coronal sections using a mouse brain slice matrix. The slices were stained with 2% 2,3,5-triphenyltetrazolium chloride (TTC; Sigma-Aldrich, St. Louis, MO) in PBS to visualize the infarctions [34]. Planimetric measurements (ImageJ software, National Institutes of Health, Bethesda, MD) blinded to the treatment groups were used to calculate lesion volumes, which were corrected for brain edema according to the following equation: V_indirect_ (mm^3^) = V_infarct_* (1 - (V_ih_ - V_ch_) / V_ch_. The term (V_ih_ - V_ch_) represents the volume difference between the ischemic hemisphere and the control hemisphere and (V_ih_ - V_ch_) / V_ch_ expresses the difference as a percentage of the control hemisphere [37,38].

### Histology

For analysis of the overall brain architecture formalin-fixed brains from naïve *hcn2*^-/-^ and *hcn2*^+/+^ littermates were embedded in paraffin-wax, cut into 5 μm thick sections and stained for cresyl violet. For hematoxylin and eosin staining *hcn2*^-/-^ and *hcn2*^+/+^ mice that underwent 30 min tMCAO were deeply anesthetized and transcardially perfused with 4% paraformaldehyde (PFA) 24 hours after reperfusion. Thereafter brains were embedded in paraffin-wax and cut into 5 μm thick sections 0.5 mm anterior from bregma (representing the ischemic territory of the MCA).

### Statistical analysis

Data are expressed as mean ± standard deviation (SD) except for the ordinal Bederson score and the grip test score that are depicted as scatter plots including median with the 25% percentile and the 75% percentile given in brackets in the text. Data were tested for Gaussian distribution with the D’Agostino and Pearson omnibus normality test and then analyzed by unpaired, 2-tailed Student’s *t*-test (infarct volumes) or the nonparametric Mann–Whitney test (Bederson score and the grip test). Real-time PCR and Laser-Doppler flowmetry data were analyzed by 1-way ANOVA in case of analyzing the interactions of two independent variables or 2-way ANOVA with post hoc Bonferroni adjustment for P values in case of multiple comparisons. *p* < 0.05 was considered statistically significant.

## Results

### *Hcn2* gene expression decreases in basal ganglia 24 h after arterial occlusion

**Figure 1:**
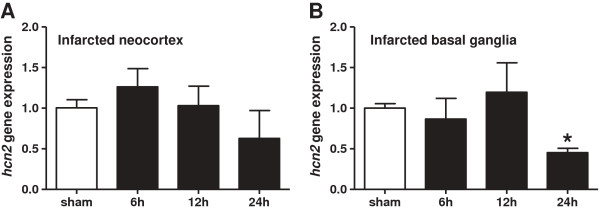
**Time course of *hcn2 *gene expression in infarcted neocortex and basal ganglia after tMCAO.** Relative *hcn2* gene expression was assessed by means of semi-quantitative real-time PCR in infarcted neocortical areas **(A)** and basal ganglia **(B)** of sham-treated WT mice as well as at three different time points after reperfusion (6 h, 12 h, 24 h). *p < 0.05, 1-way ANOVA followed by Bonferroni multiple comparison test.

It has been previously shown that mRNA expression of different cation channels is increased after MCAO in rats. This overexpression of ion channel genes can be prevented through the application of the neuroprotective substance ginsenoside-Rd [39]. Here, we tested whether transient cerebral ischemia influenced *hcn2* expression levels. Relative *hcn2* gene expression values of sham-treated WT mice in infarcted cortex (1.0 ± 0.1; Figure 1A) and basal ganglia (1.0 ± 0.1; Figure 1B) were compared with three different time points (6, 12, 24 h; n = 4-5 per group) after 60 min of tMCAO. In infarcted cortical tissue *hcn2* gene expression levels nominally peaked at 6 h after tMCAO (1.26 ± 0.2) and slightly decreased at later time points (12 h: 1.03 ± 0.2; 24 h: 0.62 ± 0.3). However, no significant differences were detected. Also in infarcted basal ganglia *hcn2* gene expression levels remained unaltered early after the insult (6 h: 0.87 ± 0.3; 12 h: 1.20 ± 0.4). However, at 24 h (0.45 ± 0.1) after tMCAO we detected a significant ~55% reduction of expression levels compared to sham-treated mice.

### Genetic ablation of HCN2 channels has no major impact on the outcome after tMCAO

**Figure 2:**
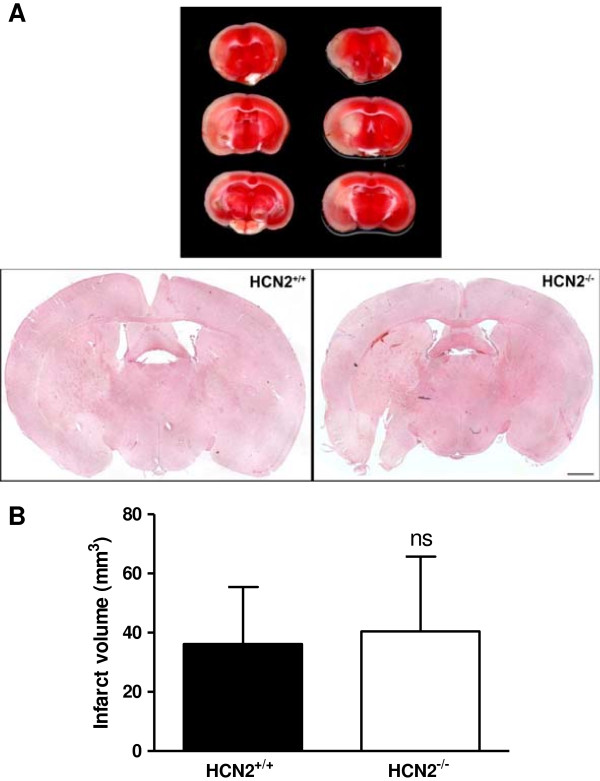
**Infarct volumes 24 h after 30 min MCA occlusion in *hcn2***^**+/+ **^**and *hcn2***^**-/- **^**mice. (A)** Representative 3,5-Triphenyltetrazoliumchloride (TTC) stained images of three corresponding coronal sections (upper panel) of control (*hcn2*^+/+^) and *hcn2*^-/-^ mice. Ischemic infarctions appear white and regularly and include the neocortex and basal ganglia as confirmed by haematoxylin and eosin staining (lower panel) (bar: 1 mm). **(B)** Mean brain infarct volumes on day1 after 30 min tMCAO in the two animal groups (*hcn2*^+/+^: n = 11; *hcn2*^-/-^ mice: n = 11). Non-significant differences (ns), unpaired, 2-tailed Student’s *t*-test.

**Figure 3:**
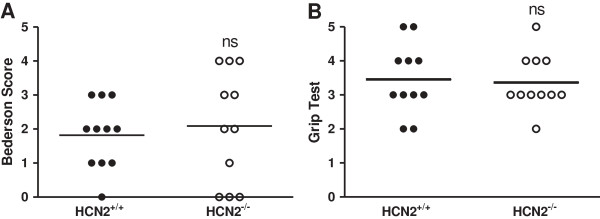
**HCN2 ablation does not affect neurological and motor functions after 30 min MCA occlusion.** Neurological Bederson score **(A)** and grip test **(B)** from the *hcn2*^+/+^ and *hcn2*^-/-^ animals shown in Figure 2 on day1 after 30 min tMCAO. Non-significant differences (ns), Mann-Whitney test.

**Figure 4:**
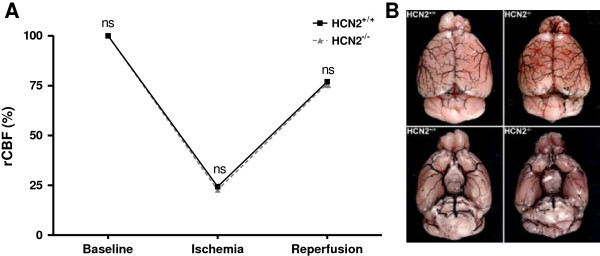
**Assessment of the cerebral vasculature in *hcn2***^**-/- **^**vs. *hcn2***^**+/+ **^**littermates. (A)** Regional cerebral blood flow (rCBF) was monitored using the Laser-Doppler flowmetry prior (baseline), during (ischemia) and shortly after (reperfusion) 60 min of tMCAO to make sure that perfusion deficits were similar in *hcn2*^-/-^ (grey) and *hcn2*^+/+^ mice (black; n = 3 per group). No differences in rCBF were observed between the groups at any time point, ns: not significant 2-way ANOVA, Bonferroni‘s post hoc test. **(B)** Assessment of the cerebral vasculature in *hcn2*^-/-^ and control (*hcn2*^+/+^) littermates by perfusion with black ink. A complete Circle of Willis was found in both animal groups and the anatomy of the MCA trunk and branch was identical in *hcn2*^+/+^ and *hcn2*^-/-^ mice.

Based on the observation that transient arterial occlusion alters *hcn2* gene expression in infarcted basal ganglia 24 h after surgery, we next investigated the functional role of HCN2 channels *in vivo* during experimental cerebral ischemia. Prior to this we found the overall brain architecture of *hcn2*^-/-^ and *hcn2*^+/+^ mice to be normal as revealed by cresyl violet staining of naïve brain sections (Additional file 1: Figure S1). Since all and 33% of HCN2-deficient and *hcn2*^+/+^ mice died after 60 min of perfusion, we induced mild ischemia by 30 min of tMCAO which resulted in a mortality of 31% in *hcn2*^+/+^ and *hcn2*^-/-^ littermates (5 mice from 16 died in each group, data not shown). 24 hours after reperfusion stroke-induced tissue damage in both groups was restricted to neocortical areas and basal ganglia as revealed by TTC-staining of 2 mm-thick coronal sections (Figure 2A). *Hcn2*^+/+^ animals showed stroke volumes of 36.2 ± 19.3 mm^3^ while *hcn2*^-/-^ mice displayed infarct areas of 40.5 ± 25.2 mm^3^ (n = 11 per group; *p* = 0.66; Figure 2B). As expected from comparable infarct sizes, no functionally relevant differences could be found for the Bederson score (median, 2.0 [1.0, 3.0] for *hcn2*^+/+^ vs. 2.0 [0.0, 4.0] for *hcn2*^-/-^; *p* = 0.64; Figure 3A) and the grip test (median, 3.0 [3.0, 4.0] for *hcn2*^+/+^ vs. 3.0 [3.0, 4.0] for *hcn2*^-/-^; *p* = 0.86; Figure 3B). As measured by laser Doppler flowmetry, no differences in cerebral perfusion between the groups at any time point could be observed (Figure 4A). Assessment of the cerebral vasculature by ink-perfusion revealed identical constitutions in *hcn2*^+/+^ and *hcn2*^-/-^ mice as a complete circle of Willis could be identified in all animals and the anatomy of the MCA trunk and branch did not differ between genotypes (Figure 4B).

## Discussion

Brain injury following transient or permanent focal cerebral ischemia develops from a complex series of pathophysiological events. Very early after the onset of a perfusion deficit membrane depolarisation in the core region of the affected brain tissue and glutamate excitotoxicity raise initial cell damage and trigger an inflammatory response as well as apoptosis in later stages of stroke development [7]. This study focuses on the impact of HCN2 channels on the early pathophysiological events after arterial occlusion. The findings of the presented study show: (1) during the course of stroke development *hcn2* gene expression remains stable in infarcted cortical areas and reveals a significant decrease in affected basal ganglia 24 h after reperfusion compared to sham-treated animals; (2) the presence of HCN2 channels does not affect stroke development regarding infarct sizes and neurological as well as motor functions assessed from tMCAO-treated mice. Although *hcn2* gene expression was found to be reduced in infarcted basal ganglia 24 h after occlusion, it is concluded that with the techniques applied in this study an impact of HCN2 channels on stroke development in mice could not be determined.

Since drugs that reduced infarct-induced depolarizations in the core and penumbra region decrease infarct sizes in rats [7,40], the prevention of membrane depolarization is regarded as one putative therapeutic strategy that intervenes stroke development in an early stage. Accordingly, ion channel function and dysfunction has been investigated intensively. In this study the time course of *hcn2* gene expression appeared inconspicuous, except a significant down regulation of about 55% in infarcted basal ganglia 24 h after reperfusion (see Figure 1). In previous studies altered gene expression levels of functionally distinct ion channels have already been observed. Overexpression of genes encoding a non-selective cation channel (TRPM7) and two sodium channels (ASIC1a, ASIC2a) was found in rat brains after 120 min of tMCAO. In that study pre-treatment with Ginsenoside-Rd, a neuroprotective substance of the Chinese herb panax ginseng, had no significant effects on the expression of TRPM1-6 and glutamate receptors but down regulated MCAO-induced expression of TRPM7, ASIC1a and ASIC2a [39]. A K^+^ leak channel of the K_2P_ family (TREK1) had a beneficial impact on primary cultured astrocytes during hypoxia. TREK1 protein expression was temporally increased in these cells at 6 h after hypoxia. However, blockade of TREK1 channels raised the number of hypoxia-induced neuronal apoptosis in a neuron-astrocyte co-culture indicating a neuroprotective role of TREK1 channels after hypoxia [41]. In line with that, former studies on K_2P_ channels in stroke also revealed that TASK1 and TREK1 are involved in neuroprotection after stroke in mice while a closely related channel (TASK3) had no influence. TREK1-deficient mice showed an increased vulnerability to ischemia because neuroprotection through polyunsaturated fatty acids (TREK1 agonists) which was impressive in wildtype animals disappeared in TREK1^-/-^ mice [4]. Similarly, the presence of functional TASK1 channels was beneficial during stroke in mice since TASK1^-/-^ mice developed significantly larger infarct volumes accompanied by worse outcome in functional neurological tests compared to wildtype mice [3]. Interestingly, TASK3 channels which are functionally closely related to TASK1 did not affect the outcome after cerebral ischemia [2].

The membrane potential is shaped by the interplay of HCN channels with inwardly rectifying K^+^ channels and K^+^ leak channels in mouse cortical [42] and by HCN2/TASK3 interactions in rat thalamocortical neurons [14]. Thus, we here tested whether HCN2 ablation would interfere with the stability of the membrane potential and thereby influence stroke pathophysiology. The present findings indicate that HCN2 channels affect neither infarct sizes (see Figure 2) nor neurological and motor functions (see Figure 3). HCN channel activation upon hyperpolarization is an unique characteristic of all HCN channels [22-24] thereby outbalancing a hyperpolarized membrane potential (<-80 mV) towards resting values (-60 to -70 mV). Moreover, these channels are sensitive to acidification [20,21] as taking place in ischemic tissue [43]. However, especially the response of I_h_ upon hypoxia is well investigated in neurons of the thalamus [44,45]. During acute thalamic hypoxia an increased release of noradrenaline, serotonin, histamine and nitric oxide is responsible for transforming I_h_ into an instantaneously activating current. These studies demonstrate that it is rather the massive release of monoaminergic transmitters than tissue acidification that influences I_h_. This I_h_ activation promotes membrane depolarization and finally neuronal damage. Since ischemia also induces release of monoamines in the basal ganglia [46], it can be assumed that HCN channels are also activated by the respective signaling pathways in these brain regions. However, HCN2 ablation in this study does not affect the extent of neuronal damage compared to control. Besides HCN2 channels HCN4 channels are highly while HCN1 and HCN3 are less sensitive to changes in intracellular cAMP [47,48]. Thus, it could be worth investigating the role of other HCN family members, presumably HCN4, in the context of ischemia in future studies.

## Competing interests

The authors declare that they have no competing interests.

## Authors’ contributions

All authors have read and approved the manuscript. PE and EG wrote the manuscript and performed the experiments. CK, TB, SB, AL and SGM conceived the experiments, analysed data, and funded the project.

## Supplementary Material

Additional file 1: Figure S1 Overall brain architecture of *hcn2*^+/+^ vs. *hcn2*^-/-^ littermates. Independent of HCN2 ablation brain architecture of both groups is found to be normal as assessed by cresyl violet staining of 5 μm thick coronal brain sections of naïve *hcn2*^+/+^ and *hcn2*^-/-^ littermates (bar: 1 mm). Click here for file
