# Supplementary material for: Ischemia-induced cell depolarization: does the hyperpolarization-activated cation channel HCN2 affect the outcome after stroke in mice?
Source: Exp Transl Stroke Med. 2013 Dec 27;5:16. doi: 10.1186/2040-7378-5-16 (PMC3879998; doi:10.1186/2040-7378-5-16)
Supplement: Additional file 1: Figure S1 — Overall brain architecture of hcn2+/+ vs. hcn2-/- littermates. Independent of HCN2 ablation brain architecture of both groups is found to be normal as assessed by cresyl violet staining of 5 μm thick coronal brain sections of naïve hcn2+/+ and hcn2-/- littermates (bar: 1 mm). [file 2040-7378-5-16-S1.pdf]

Suppl. Fig. 1

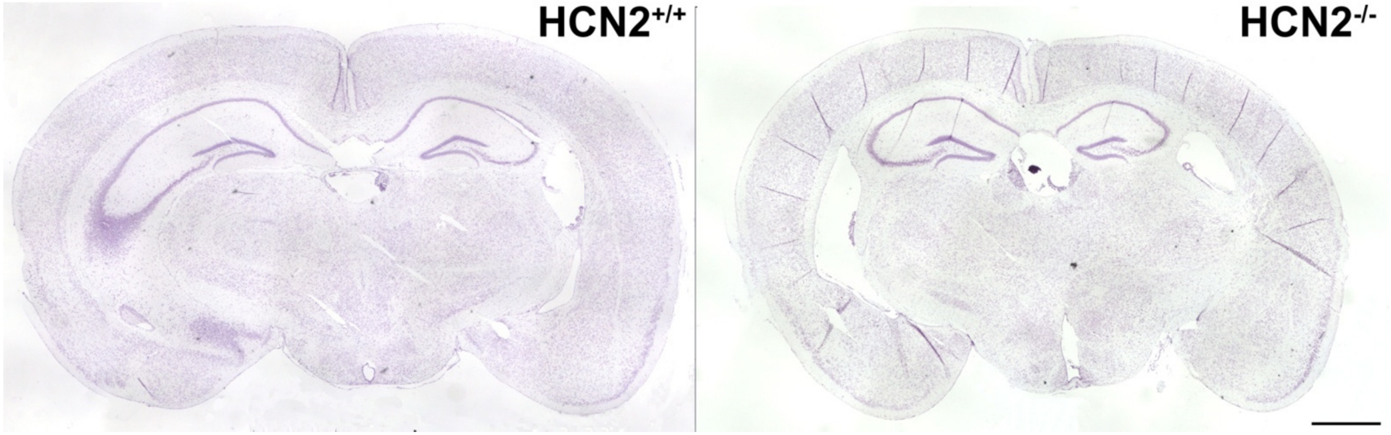

Supplementary Figure 1 - Overall brain architecture of $HCN2^{-/-}$ vs. $HCN2^{+/+}$ littermates. Independent of HCN2 ablation brain architecture of both groups is found to be normal as assessed by cresyl violet staining of 5 µm thick coronal brain sections of naïve $HCN2^{+/+}$ and $HCN2^{-/-}$ littermates (bar:1 mm).
